# The Gamboa-Hoil Point for Primary Perineal Closure During Total Pelvic Exenteration

**DOI:** 10.7759/cureus.101035

**Published:** 2026-01-07

**Authors:** Sergio Isidro Gamboa-Hoil, Ricardo Gamboa-Gutiérrez, Alejandro Medina-Campos

**Affiliations:** 1 Surgical Oncology, Mexican Social Security Institute, Mérida, MEX; 2 Medical Research, Mexican Social Security Institute, Mérida, MEX

**Keywords:** colorectal cancer, empty pelvis syndrome, perineal closure, perineal morbidity, postoperative complications, surgical technique, total pelvic exenteration

## Abstract

Introduction

Total pelvic exenteration (TPE) is a radical procedure for selected patients with advanced pelvic malignancies and is associated with significant perineal morbidity. Despite multiple reconstructive strategies, no consensus exists regarding the optimal strategy for perineal closure. The Gamboa-Hoil point is a standardized primary closure technique proposed by the authors, designed to reinforce perineal wound closure using a simple suture-based approach.

Methods

We conducted a single-center, retrospective descriptive study including patients with colorectal cancer (rectal, sigmoid colon, and selected locally advanced or recurrent anal canal tumors) who underwent TPE between January 2021 and December 2024. Primary perineal closure was performed using the Gamboa-Hoil point. The primary outcome was perineal morbidity, including wound dehiscence, surgical site infection, perineal sinus formation, and reintervention.

Results

Eleven patients (six men and five women) were included, with a median age of 60 years (range, 40-67). Negative surgical margins (R0) were achieved in all cases. No cases of infected pelvic fluid collections or postoperative intestinal obstruction were observed. A urinary fistula occurred in one patient (9.1%) and required minor reintervention with drain repositioning. Perineal wound dehiscence occurred in one patient (9.1%), and persistent perineal sinus formation was observed in two patients (18.2%). The median length of hospital stay was seven days. During a median follow-up of 20 months, tumor recurrence occurred in two patients (18.2%). Overall survival at study closure was 72.7%.

Conclusion

The Gamboa-Hoil point appears to be a feasible and reproducible option for primary perineal closure following TPE, with acceptable perineal morbidity in patients in whom primary closure is feasible.

## Introduction

Total pelvic exenteration, originally described by Brunschwig [1], is a radical surgical procedure involving en bloc removal of the sigmoid colon, rectum, and anus. In male patients, the operation additionally entails resection of the bladder, seminal vesicles, prostate, and urethra, whereas in female patients it may include en bloc resection of the uterus ± adnexa, vagina, bladder, and urethra, depending on oncologic requirements [2].

This procedure is indicated in patients with locally advanced primary or recurrent pelvic malignancies with the aim of achieving complete oncologic resection with negative margins (R0). Although associated with substantial functional impact, total pelvic exenteration has been linked to a modest improvement in overall survival in carefully selected patients [2].

Empty pelvis syndrome (EPS)

EPS, first described in 1993, refers to a spectrum of postoperative complications after total pelvic exenteration, including pelvic collections, bowel obstruction, perineal sinus formation, and fistula development [3]. These events are largely related to the creation of a large pelvic cavity after multivisceral resection. EPS has been reported in 32.1% of patients, with 17.7% diagnosed during the index hospitalization and 14.4% after hospital discharge. Additionally, 5.2% of patients require delayed reintervention (>90 days) due to EPS-related complications [4].

Perineal closure techniques

Reconstructive strategies aimed at managing the perineal defect and pelvic anatomy following total pelvic exenteration have shown heterogeneous outcomes, with no clear superiority of a single approach [3]. Several studies and systematic reviews have reported increased perioperative morbidity associated with more extensive perineal reconstruction, including higher blood loss, prolonged hospitalization, and wound-related complications, without consistent oncologic benefit when compared with primary perineal closure [4-7].

In Mexico, early institutional experience reported by Luna-Perez et al. [8] in a tertiary referral center described the use of primary perineal closure with simple nylon sutures in selected patients undergoing total pelvic exenteration, in some cases combined with adjunctive measures such as omentoplasty or temporary perineal packing. This experience highlights the historical feasibility of simple suture-based approaches for perineal defect management and provides a conceptual framework for the development of standardized, anatomically guided techniques.

Rationale for the Gamboa-Hoil point

Although no definitive technique exists for pelvic reconstruction after total pelvic exenteration, current evidence suggests that perineal closure strategies should be individualized according to patient characteristics, extent of resection, and institutional resources [4]. This is particularly relevant in settings with limited access to reconstructive or plastic surgical support, where the oncologic surgeon frequently performs the entire procedure, including perineal closure.

The surgical technique described in this study is not intended to eliminate the pelvic dead space nor to treat empty pelvis syndrome itself. Instead, the Gamboa-Hoil point is designed to reinforce primary perineal closure, with the specific aim of reducing wound dehiscence and secondary infection, without increasing the incidence of major pelvic complications such as bowel obstruction or deep pelvic collections.

In this context, the relevance of EPS in the present study lies in demonstrating that the use of a simplified anchoring perineal closure technique does not exacerbate overall pelvic morbidity following total pelvic exenteration. In patients in whom primary closure is feasible, the Gamboa-Hoil point represents a simple and reproducible alternative to flap-based reconstruction, avoiding the need for plastic surgery while maintaining acceptable perineal outcomes.

Accordingly, the Gamboa-Hoil point may be considered a feasible option for primary perineal closure in patients undergoing total pelvic exenteration. Therefore, the aim of this study was to describe the clinical outcomes and perineal morbidity associated with the use of the Gamboa-Hoil point for primary perineal closure in patients with colorectal cancer, including rectal, sigmoid colon, and selected locally advanced or recurrent anal canal tumors, undergoing total pelvic exenteration.

## Materials and methods

This was a single-center, retrospective descriptive study.

Study population

This study was approved by the Ethics and Research Committee of our institution. Due to the retrospective nature of the study, the requirement for written informed consent was waived.

Between January 2021 and December 2024, a total of 11 patients with colorectal cancer, including rectal cancer, sigmoid colon cancer, and selected locally advanced or recurrent anal canal tumors requiring total pelvic exenteration due to adjacent organ invasion, underwent surgery at our tertiary oncology referral center. All patients who met the predefined inclusion criteria were consecutively enrolled.

In this series, the Gamboa-Hoil point was systematically used as the primary perineal closure technique in all patients undergoing total pelvic exenteration. The technique is intended for cases in which the perineal defect allows primary closure in a single stage without extensive skin loss, which was the situation in all included patients.

In all cases, primary perineal closure was performed using the Gamboa-Hoil point as the sole perineal closure technique.

Exclusion criteria included partial pelvic exenteration, abdominoperineal resection without total pelvic exenteration, the use of flap-based or plastic surgical perineal reconstruction, incomplete clinical records, and insufficient postoperative follow-up to allow adequate assessment of perineal outcomes.

Surgical technique

All procedures were performed by a single board-certified surgical oncologist, assisted by a general surgeon and/or general surgery residents.

During the first operative phase, patients were positioned in the lithotomy position, and a midline supraumbilical and infraumbilical incision was performed. After systematic exploration of the abdominal cavity to exclude distant disease, the mesocolon was incised at its root. The inferior mesenteric artery was ligated at its origin, followed by ligation of the inferior mesenteric vein as indicated.

Total pelvic exenteration was performed according to oncologic requirements. In male patients, the procedure included en bloc resection of the prostate and seminal vesicles. In female patients, the procedure included en bloc hysterectomy; bilateral salpingo-oophorectomy was not performed in this cohort. In all cases, the urinary bladder was resected as part of the exenterative procedure.

Both ureters were identified and ligated proximally to the tumor, and pelvic dissection was completed to the pelvic floor with total mesorectal excision when applicable.

During the second operative phase, a perineal incision was performed using the ischial tuberosities, coccyx, and the midline of the perineum as anatomical landmarks. Complete mobilization of the rectum was then achieved through anterior, posterior, and lateral dissection. Following completion of the resection, primary perineal closure was performed using the Gamboa-Hoil point, a standardized perineal closure technique, which is described in detail below.

Description of the Gamboa-Hoil point

Primary perineal closure was performed using a nonabsorbable multifilament polyester suture (Ethibond® EXCEL, size 5 (metric 7.0), 75-cm suture length, taper-cut needle).

After irrigation of the perineal wound, the Gamboa-Hoil point was applied using three sequential deep suture layers placed in a stepwise manner to reinforce deep perineal anchorage and achieve uniform tension distribution across the perineal closure.

For each suture layer, the stitch was placed in two sequential steps. The first pass was performed from the left lateral perineum toward the midline (lateral-to-medial), exiting at the perineal midline. The second pass continued from the midline toward the contralateral perineum (medial-to-lateral), completing the anchoring layer with symmetric tissue capture.

These layers are placed in a graduated fashion, with progressively decreasing depth and lateral distance from the perineal skin edge. The first layer is positioned at a depth and lateral distance greater than 3 cm from the perineal margin, the second at greater than 2 cm, and the third at greater than 1 cm. This configuration provides deep perineal anchorage and progressive tension redistribution, contributing to stabilization of the perineal closure without attempting to obliterate the pelvic cavity.

**Figure 1:**
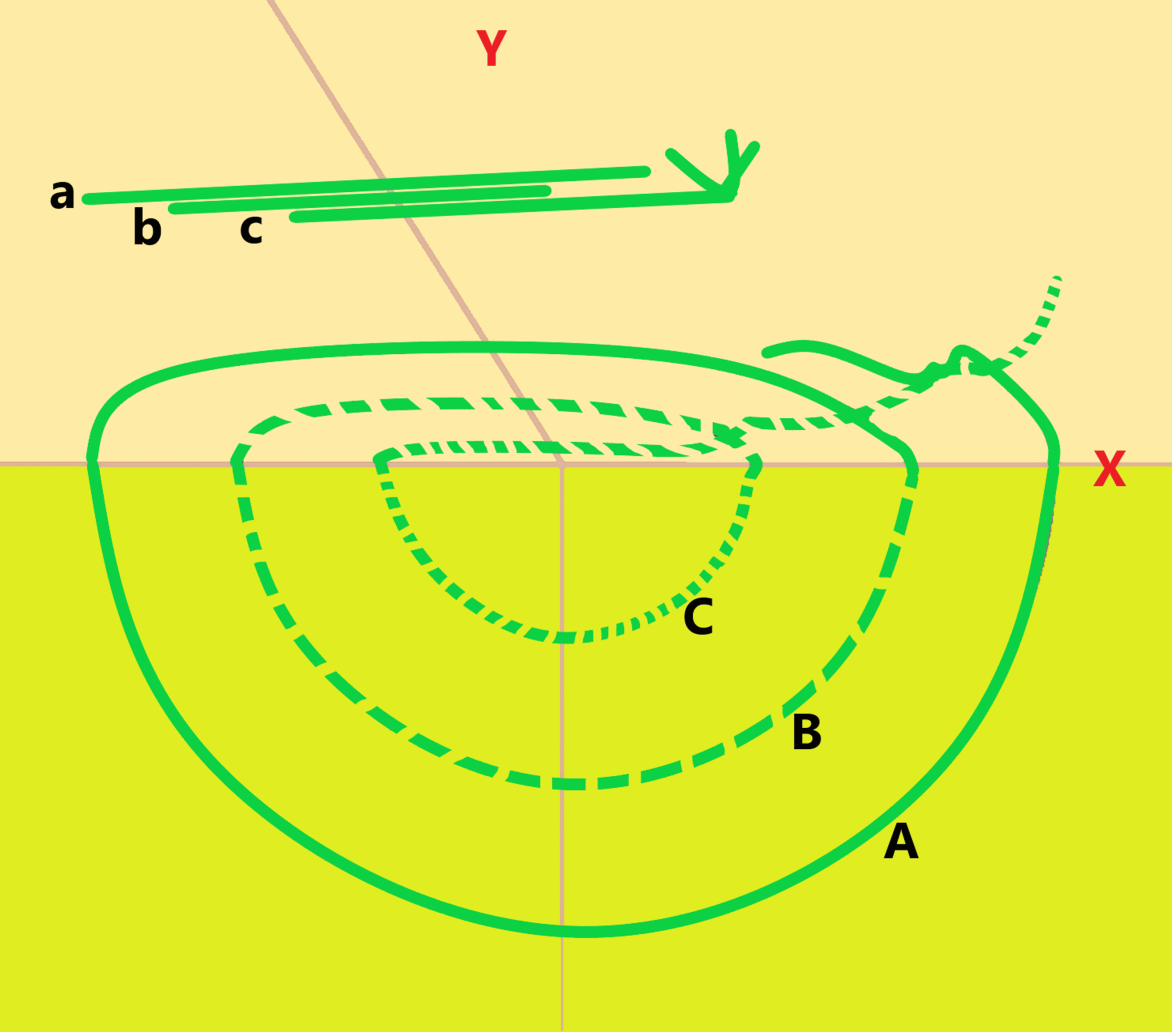
Schematic Representation of the Gamboa-Hoil Point for Primary Perineal Closure The perineal region is located above the horizontal reference line (X), while the pelvic cavity is located below it. The schematic illustrates the placement of three sequential deep anchoring suture layers arranged concentrically to achieve deep perineal fixation and progressive tension redistribution across the perineal closure. The suture layers are placed in a graduated fashion, with progressively decreasing depth and lateral distance from the perineal skin edge. The first layer (A) is positioned at a depth and lateral distance greater than 3 cm from the perineal margin (far-far), the second layer (B) at greater than 2 cm (intermediate-intermediate), and the third layer (C) at greater than 1 cm (near-near). For each suture layer, the first needle pass is performed from the left lateral perineum toward the midline (lateral-to-medial), exiting at the perineal midline. The second pass then continues from the midline toward the contralateral perineum (medial-to-lateral), completing the anchoring layer with symmetric tissue capture. Additional Gamboa-Hoil points are placed at intervals of approximately 1.5-2 cm along the perineal incision to ensure uniform support throughout the closure. Green lines represent the Ethibond® sutures used to construct the three-layer Gamboa-Hoil point. Variations in line thickness are intentionally used to differentiate overlapping suture trajectories and depth planes. Uppercase letters (A-C) denote the depth of the anchoring suture layers, whereas lowercase letters (a-c) represent the corresponding superficial trajectory of a separate Gamboa-Hoil point depicted within the same schematic. Image created by the author.

These three layers correspond to progressively superficial planes, commonly referred to as a “far-far,” “intermediate-intermediate,” and “near-near” configuration, which allows gradual tension redistribution and secure perineal anchorage. Figure 1 illustrates the stepwise placement and depth of each suture layer.

Subsequent Gamboa-Hoil points were spaced approximately 1.5-2 cm apart along the perineal incision, providing uniform support across the closure.

During the third operative phase, an ileal conduit was constructed using a 40-cm segment of ileum located approximately 50 cm proximal to the ileocecal valve. Ureteral stents were placed bilaterally and secured to the ileal conduit, which was then exteriorized through the right hemiabdomen.

During the fourth operative phase, a pelvic drain was placed, followed by layered closure of the abdominal wall and creation of a terminal colostomy in the left hemiabdomen [2].

Postoperative management

Routine postoperative admission to the intensive care unit (ICU) was not required. Octreotide was not administered, and nasogastric tube placement was not routinely used. Oral intake was initiated on postoperative day 3.

A closed-suction drain (Drenovac®) was exteriorized through the right hemiabdomen and directed to the pelvic cavity, where it was maintained for four weeks.

Outcome variables

The primary outcome was perineal morbidity related to primary perineal closure following total pelvic exenteration, including wound dehiscence, surgical site infection, perineal sinus formation, and the need for reintervention related to the perineal wound.

Statistical analysis

Statistical analyses were performed using IBM SPSS Statistics, Version 25.0 (IBM Corp., Armonk, NY). A descriptive statistical analysis was conducted using measures of central tendency and dispersion. Quantitative variables are presented as mean and standard deviation or as median and interquartile range, depending on data distribution. Categorical variables are reported as absolute frequencies and percentages. Given the descriptive nature of the study, the small sample size, and the absence of a comparison group, no inferential statistical analyses or between-group comparisons were performed.

## Results

Between January 2021 and December 2024, a total of 11 patients (six men and five women) underwent total pelvic exenteration at our institution. All procedures were performed by a single board-certified surgical oncologist, with assistance from a general surgeon and/or general surgery residents.

The median age of the study population was 60 years (range, 40-67 years). Primary rectal cancer was the most frequent indication for surgery, accounting for 81.8% of cases (n = 9). Neoadjuvant chemotherapy and radiotherapy were administered to six patients (54.5%), in accordance with standard oncologic protocols; omission of neoadjuvant treatment was related to locally advanced or recurrent disease with urinary bladder involvement, particularly extension to the vesical trigone, requiring upfront surgical management. Adenocarcinoma was the predominant histological subtype, observed in 10 patients (90.9%). The median tumor size was 5.5 cm (range, 4.0-6.0 cm). Baseline clinical and tumor characteristics are summarized in Table 1.

**Table 1:** Baseline Clinical and Tumor Characteristics of the Study Population Data are presented as number (%) unless otherwise specified. Tumor size is reported as median and interquartile range (IQR). Preoperative organ involvement was determined based on preoperative clinical assessment and imaging findings.

Characteristics	
Sex, n (%)	
Male	6 (54.5)
Female	5 (45.5)
Comorbidities, n (%)	
Diabetes mellitus	4 (36.4)
Hypertension	2 (18.2)
Tumor location, n (%)	
Rectum	9 (81.8)
Sigmoid colon	1 (9.1)
Anal canal	1 (9.1)
Tumor status, n (%)	
Primary	9 (81.8)
Rectum	8 (88.9)
Anal canal	1 (11.1)
Recurrent	2 (18.2)
Rectum	1 (50)
Sigmoid colon	1 (50)
Neoadjuvant chemotherapy and radiotherapy, n (%)	6 (54.5)
Histology, n (%)	
Adenocarcinoma	10 (90.9)
Squamous cell carcinoma	1 (9.1)
Preoperative organ involvement, n (%)	
Rectum, prostate, and bladder	5 (45.5)
Rectum, vagina, and bladder	5 (45.5)
Colon, prostate, and bladder	1 (9.1)
Tumor size, cm, median (IQR)	5.5 (4.0-6.0)

The median intraoperative blood loss was 650 mL (range, 500-700 mL), and the median number of transfused packed red blood cell units was 2 (range, 1-2). A complete pathological response was observed in one patient (9.1%), and negative surgical margins (R0) were achieved in all cases. Surgical and pathological characteristics are summarized in Table 2.

**Table 2:** Surgical, Pathological, and Postoperative Outcomes Data are presented as n (%) unless otherwise specified. Continuous variables are reported as median (interquartile range) or median (range), as indicated. Pathological staging was reported according to the American Joint Committee on Cancer (AJCC)/Union for International Cancer Control (UICC) staging system. The prefix “yp” denotes post-neoadjuvant pathological staging.

Surgical characteristics	
Intraoperative blood loss, mL, median (IQR)	650 (500-700)
Transfused packed red blood cell units, median (IQR)	2 (1-2)
Pathological outcomes	
Complete pathological response, n (%)	1 (9.1)
Negative surgical margins (R0)	11 (100)
Pathological T stage, n (%)	
ypT0	1 (9.1)
pT4a	1 (9.1)
pT4b	9 (81.8)
Pathological N stage, n (%)	
pN0	9 (81.8)
pN1a	1 (9.1)
pN1b	1 (9.1)
Pathological M stage, n (%)	
M0	10 (90.9)
M1	1 (9.1)
Oncologic category, n (%)	
IIc	7 (63.6)
IIIb	1 (9.1)
IVa	1 (9.1)
Local recurrence	2 (18.2)
Lymphovascular invasion, n (%)	1 (9.1)
Perineural invasion, n (%)	1 (9.1)
Postoperative complications	
Infected fluid collections, n (%)	0 (0)
Intestinal obstruction, n (%)	0 (0)
Urinary fistula, n (%)	1 (9.1)
Perineal sinus, n (%)	2 (18.2)
Perineal wound dehiscence, n (%)	1 (9.1)
Reintervention, n (%)	1 (9.1)
Hospital stay	
Length of hospital stay, days, median (range)	7 (7-15)

Oncologic treatment strategy

According to standard oncologic practice, patients with primary rectal cancer are typically treated with neoadjuvant chemoradiotherapy. In this series, patients with primary rectal cancer who presented with locally advanced disease involving adjacent organs, particularly invasion of the urinary bladder with extension to the vesical trigone, were managed with upfront total pelvic exenteration. In these cases, trigonal involvement was associated with recurrent urinary tract infections and urinary obstruction, which precluded the safe administration of neoadjuvant chemotherapy or radiotherapy.

Patients with recurrent rectal cancer had previously received chemoradiotherapy followed by low anterior resection with colorectal anastomosis. During surveillance, local recurrence with invasion of the urinary bladder, including the vesical trigone, was identified, and these patients were subsequently treated with total pelvic exenteration.

The patient with sigmoid colon cancer had initially undergone anterior resection followed by adjuvant chemotherapy. During follow-up, local recurrence with bladder invasion extending to the vesical trigone was detected, and total pelvic exenteration was indicated.

Anal canal cancer is generally treated with definitive chemoradiotherapy. However, in this cohort, one patient presented with a bulky primary disease complicated by a rectovesical fistula and direct invasion of the urinary bladder and trigone, which necessitated primary surgical management with total pelvic exenteration.

Across these scenarios, invasion of the urinary bladder, particularly involvement of the vesical trigone, and recurrent urinary tract infections were key factors that limited the use of chemoradiotherapy and prompted upfront exenterative surgical management. Individual patient-level clinical, oncologic, and perineal outcomes are summarized in the Appendices.

Postoperative complications

Postoperative morbidity was acceptable. No patients developed infected fluid collections or postoperative intestinal obstruction. A urinary fistula occurred in one patient (9.1%), requiring a minor reintervention consisting solely of drain repositioning. Perineal wound dehiscence was observed in one patient (9.1%), who subsequently developed a perineal sinus. Overall, perineal sinus formation occurred in two patients (18.2%).

Adjuvant treatment

Adjuvant systemic therapy was administered to seven patients (63.6%). No patients received adjuvant radiotherapy. Adjuvant treatment characteristics are summarized in Table 3. Adjuvant systemic therapy was not administered in selected cases due to poor postoperative performance status and delayed postoperative recovery, which precluded the timely initiation of adjuvant therapy.

**Table 3:** Adjuvant Treatment After Total Pelvic Exenteration Data are presented as n (%). Adjuvant treatment decisions were based on postoperative clinical status and multidisciplinary oncologic assessment.

Treatment	
Adjuvant chemotherapy, n (%)	
Yes	7 (63.6)
No	4 (36.4)
Adjuvant radiotherapy, n (%)	
No	11 (100)

Recurrence

The median follow-up period was 20 months (range, four to 45 months). During follow-up, tumor recurrence was observed in two patients (18.2%), all of whom belonged to the primary tumor group. Tumor recurrence patterns are summarized in Table 4.

**Table 4:** Recurrence and Survival Outcomes Data are presented as n (%) unless otherwise specified. Time to recurrence is reported in months.

Outcomes	
Tumor recurrence, n (%)	
Yes	2 (18.2)
No	9 (81.8)
Recurrence site, n (% of patients with recurrence)	
Local + inguinal	2 (100)
Time to recurrence, months, n	
6	1
7	1
Overall survival at study closure, n (%)	
Alive	8 (72.7)

Overall survival

At the end of the follow-up period, eight patients (72.7%) were alive. No deaths were recorded among patients who developed tumor recurrence.

## Discussion

The median age of our study population and the predominance of male sex were comparable to those reported by Vigneswaran et al. (median age, 59 years; 53% male) [9]. Although the proportion of patients with diabetes mellitus in our cohort was higher than that reported in their series (10.9%), this finding may reflect the high prevalence of metabolic comorbidities in our regional population. Regarding tumor distribution, rectal cancer accounted for 81% of cases, followed by sigmoid colon cancer in 9.1% and anal canal cancer in 9.1%, a pattern consistent with previous reports identifying rectal tumors as the most frequent indication for total pelvic exenteration. In the patient with sigmoid colon cancer, the indication for exenterative surgery was related to locally recurrent disease with deep pelvic extension and invasion of adjacent organs, including the urinary bladder with extension to the vesical trigone, rather than primary tumor location alone.

Adenocarcinoma was the predominant histological subtype in our cohort, consistent with findings reported in the literature. Direct comparisons, however, are limited, as most published series predominantly include patients with colon and rectal cancers. In our population, 81% of cases presented with locally advanced disease, a proportion comparable to that reported by Katory et al. [10] (85.7%), who identified advanced disease as a major indication for exenterative surgery.

Neoadjuvant combined chemotherapy and radiotherapy were administered to 54.5% of patients in our study, a lower proportion than that reported by Pokharkar et al. [11], in whose series all patients received neoadjuvant treatment, likely reflecting differences in institutional protocols and patient selection.

In a previously published series of total pelvic exenteration, histologically confirmed invasion of adjacent pelvic organs has been reported in approximately 64-66% of cases. In contrast, our cohort demonstrated multivisceral involvement, most commonly affecting the rectum, bladder, prostate, and vagina. These findings underscore the high burden of locally advanced disease in our population and are consistent with the advanced oncologic stage typically required to justify total pelvic exenteration [8].

The median intraoperative blood loss observed in our cohort was 650 mL, which is lower than that reported in several previously published series of total pelvic exenteration for colorectal malignancies. In the literature, reported median or mean blood loss values generally range from approximately 1,000 mL to more than 2,000 mL [11-15].

It is noteworthy that one patient (9.1%) achieved a complete pathological response following surgical resection. In our cohort, negative surgical margins (R0) were achieved in 100% of cases, a finding that is consistent with reports in the literature for primary locally advanced rectal cancer [11,16]. In contrast, series evaluating total pelvic exenteration performed for recurrent pelvic malignancies have reported lower R0 rates, ranging from 57% to 76% [14,17].

Overall postoperative morbidity was acceptable. No cases of infected pelvic fluid collections or postoperative intestinal obstruction were observed, findings that may be influenced by perioperative management strategies such as prolonged pelvic drainage and antibiotic coverage rather than the perineal closure technique itself. Urinary fistula occurred in one patient (9.1%), a rate higher than that reported in the literature [4]; however, this complication was not directly related to the perineal closure technique. Persistent perineal sinus formation was observed in two patients (18.2%).

Perineal wound dehiscence occurred in one patient (9.1%), a rate that compares favorably with those reported in the literature, where dehiscence rates of up to 26.7% have been described in patients undergoing complex perineal reconstruction [5].

The median length of hospital stay in our cohort was 7 days, which is shorter than that reported in the literature, where median hospital stays after total pelvic exenteration typically range from 10 to 19 days [9-11,15].

With a median follow-up of 20 months, the recurrence rate observed in our study was 18.2%, which is higher than that reported by Miri et al. [18] (9.1%). In terms of overall survival, 72.7% of patients in our cohort were alive at the end of the study period, a rate comparable to the one-year overall survival of approximately 80% reported by Miri et al. [18].

Total pelvic exenteration is reserved for selected patients with locally advanced or recurrent pelvic malignancies requiring en bloc resection of multiple pelvic organs. In this context, perineal reconstruction aims primarily to prevent small bowel evisceration and perineal wound dehiscence. The Gamboa-Hoil point was developed as a simplified, suture-based primary closure strategy for patients in whom primary closure is feasible, without the need for additional reconstructive procedures.

Taken together, these findings suggest that the Gamboa-Hoil point represents a feasible and reproducible option for primary perineal closure following total pelvic exenteration, with the potential to reduce perineal wound morbidity in patients in whom primary closure is feasible.

Limitations

This study has several limitations that should be acknowledged. First, its retrospective and descriptive design, combined with the small sample size, limits the ability to perform inferential statistical analyses or establish causal relationships. Second, the study was conducted at a single center, and all procedures were performed by a single experienced surgical oncologist, which may limit the generalizability of the results to other institutions with different levels of expertise.

Third, the absence of a comparison group precludes direct comparison between the Gamboa-Hoil point and other perineal closure techniques, including myocutaneous flaps or mesh-based reconstruction. Therefore, no conclusions can be drawn regarding superiority or equivalence with more complex reconstructive approaches. Additionally, the duration of follow-up was relatively limited for assessing long-term oncologic outcomes, particularly overall survival and late perineal complications.

Finally, selection bias cannot be excluded, as only cases in which primary perineal closure was feasible were included. All patients underwent total pelvic exenteration and were managed with the same perineal closure technique, which limits the generalizability of the findings to patients requiring extensive perineal skin resection or flap-based reconstruction. Importantly, this study was not designed to evaluate interventions aimed at obliterating pelvic dead space or treating the empty pelvis syndrome. This work is intended as a technical description of a primary perineal closure option rather than a comparative evaluation of reconstructive strategies. Larger, multicenter prospective studies with comparative designs are needed to further evaluate the effectiveness and reproducibility of this perineal closure technique.

## Conclusions

In this retrospective descriptive study, the Gamboa-Hoil point was found to be a feasible and reproducible technique for primary perineal closure following total pelvic exenteration in patients with colorectal cancer. The technique was associated with acceptable perineal morbidity and low rates of wound dehiscence, without an apparent increase in procedure-related complications.

Although these findings should be interpreted with caution due to the study’s limitations, the Gamboa-Hoil point may represent a simple and standardized option for perineal closure, particularly in settings with limited access to complex reconstructive procedures. Further prospective and comparative studies are warranted to validate these results and to better define the role of this technique in broader clinical practice.

## Appendices

**Table 5:** Individual Patient Characteristics, Tumor Features, and Perineal Outcomes After Total Pelvic Exenteration yp indicates pathological staging after neoadjuvant therapy; pT indicates pathological staging without neoadjuvant treatment.

Patient number	Sex	Age	Tumor location	Primary vs. recurrent	Neoadjuvant therapy (yes/no)	Histology	Pathological stage	Perineal wound dehiscence	Perineal sinus
1	M	66	Rectum	Primary	Yes	Adenocarcinoma	ypT0	No	Yes
2	F	69	Rectum	Recurrent	Yes	Adenocarcinoma	ypT4b	No	No
3	M	75	Rectum	Primary	No	Adenocarcinoma	pT4b	No	No
4	M	40	Sigmoid	Recurrence	No	Adenocarcinoma	pT4b	Yes	Yes
5	F	35	Rectum	Primary	Yes	Adenocarcinoma	ypT4b	No	No
6	M	60	Rectum	Primary	No	Adenocarcinoma	pT4b	No	No
7	F	35	Anal canal	Primary	No	Squamous cell carcinoma	pT4	No	No
8	M	65	Rectum	Primary	Yes	Adenocarcinoma	ypT4b	No	No
9	F	59	Rectum	Primary	Yes	Adenocarcinoma	ypT4b	No	No
10	M	67	Rectum	Primary	Yes	Adenocarcinoma	ypT4b	No	No
11	F	40	Rectum	Primary	No	Adenocarcinoma	pT4b	No	No

## References

[1] Brunschwig A (1948). Complete excision of pelvic viscera for advanced carcinoma; a one-stage abdominoperineal operation with end colostomy and bilateral ureteral implantation into the colon above the colostomy. Cancer.

[2] Grimes WR, Dunton CJ, Stratton M (2024). Pelvic exenteration. StatPearls.

[3] PelvEx Collaborative (2024). The empty pelvis syndrome: a core data set from the PelvEx collaborative. Br J Surg.

[4] West CT, Tiwari A, Smith J, Yano H, West MA, Mirnezami AH (2025). Empty pelvis syndrome as a cause of major morbidity after pelvic exenteration: validation of a core data set. Br J Surg.

[5] Bercz A, Alvarez J, Rosen R (2025). Assessment of morbidity and predictors of wound complications following perineal wound closure after radical anorectal oncologic resection: retrospective cohort study. BJS Open.

[6] Johnson YL, West MA, Gould LE (2022). Empty pelvis syndrome: a systematic review of reconstruction techniques and their associated complications. Colorectal Dis.

[7] Riva CG, Kelly ME, Vitellaro M (2023). A comparison of surgical techniques for perineal wound closure following perineal excision: a systematic review and network meta-analysis. Tech Coloproctol.

[8] Luna-Perez P, Delgado S, Labastida S, Ortiz N, Rodriguez D, Herrera L (1996). Patterns of recurrence following pelvic exenteration and external radiotherapy for locally advanced primary rectal adenocarcinoma. Ann Surg Oncol.

[9] Vigneswaran HT, Schwarzman LS, Madueke IC, David SM, Nordenstam J, Moreira D, Abern MR (2021). Morbidity and mortality of total pelvic exenteration for malignancy in the US. Ann Surg Oncol.

[10] Katory M, McLean R, Paez E, Kucukmetin A, Naik R (2017). Short- and long-term outcomes following pelvic exenteration for gynae-oncological and colorectal cancers: a 9 year consecutive single-centre cohort study. Int J Surg.

[11] Pokharkar A, Kammar P, D'souza A, Bhamre R, Sugoor P, Saklani A (2018). Laparoscopic pelvic exenteration for locally advanced rectal cancer, technique and short-term outcomes. J Laparoendosc Adv Surg Tech A.

[12] Mehta AM, Hellawell G, Burling D, Littler S, Antoniou A, Jenkins JT (2018). Transperineal retropubic approach in total pelvic exenteration for advanced and recurrent colorectal and anal cancer involving the penile base: technique and outcomes. Tech Coloproctol.

[13] Ike H, Shimada H, Yamaguchi S, Ichikawa Y, Fujii S, Ohki S (2003). Outcome of total pelvic exenteration for primary rectal cancer. Dis Colon Rectum.

[14] Ghouti L, Pereira P, Filleron T, Humeau M, Guimbaud R, Selves J, Carrere N (2015). Pelvic exenterations for specific extraluminal recurrences in the era of total mesorectal excision: is there still a chance for cure?: a single-center review of patients with extraluminal pelvic recurrence for rectal cancer from March 2004 to November 2010. Am J Surg.

[15] Sardi A, Bolton JS, Hicks TC, Skenderis BS 2nd (1994). Total pelvic exenteration with or without sacral resection in patients with recurrent colorectal cancer. South Med J.

[16] Chen HS, Sheen-Chen SM (2001). Total pelvic exenteration for primary local advanced colorectal cancer. World J Surg.

[17] Rombouts AJ, Koh CE, Young JM (2015). Does radiotherapy of the primary rectal cancer affect prognosis after pelvic exenteration for recurrent rectal cancer?. Dis Colon Rectum.

[18] Miri SR, Akhavan S, Mousavi AS (2022). A systematic review on overall survival and disease-free survival following total pelvic exenteration. Asian Pac J Cancer Prev.

